# Atypical catabolite repression of Geobacillus kaustophilus iol operons in the presence of ribose

**DOI:** 10.1099/mic.0.001702

**Published:** 2026-04-28

**Authors:** Ken-ichi Yoshida, Kaho Fukui, Takuma Osawa, Moeka Tsuji, Miyuki Kado-Matsushita, Nao Asahi, Shu Ishikawa, Yuh Shiwa, Hirofumi Yoshikawa

**Affiliations:** 1 Department of Science, Technology and Innovation, Kobe University, 1-1 Rokkodai, Nada, Kobe 657-8501, Japan; 2 Department of Molecular Microbiology, Tokyo University of Agriculture, 1-1-1 Sakuragaoka, Setagaya, Tokyo 156-8502, Japan; 3 Genome Research Center, NODAI Research Institute, Tokyo University of Agriculture, 1-1-1 Sakuragaoka, Setagaya, Tokyo 156-8502, Japan; 4 Department of Bioscience, Tokyo University of Agriculture, 1-1-1 Sakuragaoka, Setagaya, Tokyo 156-8502, Japan

**Keywords:** *Bacillus subtilis*, catabolite repression, *Geobacillus kaustophilus*, inositol, ribose

## Abstract

*Geobacillus kaustophilus* HTA426 is a thermophilic Gram-positive bacterium that encodes the *myo*-inositol-inducible *iol* operons, the products of which form the *myo*-inositol catabolic pathway. Strain PS8 is a mutant of HTA426 that is defective in *myo*-inositol catabolism due to constitutive repression of the *iol* operons. Several spontaneous suppressor mutants of PS8, which restored growth on *myo*-inositol, were obtained. These suppressors had mutations that may affect the translation of *crh*. Inclusion of a plasmid-based copy of *crh* into the suppressor mutants concomitantly restored the repression of the *iol* operons. PS8 and its suppressor mutants shared a mutated allele of *hprK*(G268R) that may encode a defective HPr kinase/phosphorylase, which could lead to the accumulation of the phosphorylated form of Crh. Inclusion of a plasmid-based copy of the wild-type *hprK* gene into PS8 restored the induction of the *iol* operons. Conversely, inclusion of a plasmid-based copy of *hprK*(G268R) into HTA426 significantly repressed the *iol* operons. In HTA426, ribose, rather than glucose, repressed the *iol* operons. During the growth on ribose, the expression of *ptsH*, which encodes HPr, was kept at a low level, while that of *crh* was elevated. Deletion of *hprK*, *ccpA* (encoding global regulator CcpA) or the *rbs* operon (encoding the components for ribose catabolism) abolishes the ribose-induced repression of *iol* operons. These results suggest an atypical catabolite repression of the *iol* operons of *G. kaustophilus* HTA426, where HPrK phosphorylates Crh in the presence of ribose, and the phosphorylated form of Crh cooperates with CcpA to repress the transcription of the *iol* operons.

**Impact Statement**

Our findings suggest that in *Geobacillus kaustophilus* HTA426, a previously uncharacterized form of carbon catabolite repression functions on the *iol* genes, where ribose, rather than glucose, serves as the primary repressive carbon source. This expands current models of carbon catabolite repression and underscores the role of Crh in mediating the atypical regulatory response to carbon source availability in this thermophilic Gram-positive bacterium. In addition, the amino acid substitution in HPrK that disrupts catabolite repression highlights the delicate balance of Crh phosphorylation/dephosphorylation and could open new avenues for exploring the flexibility in carbon catabolite repression in different natural environments.

## Data Summary

The authors confirm that all supporting data and protocols have been provided within the article. The raw sequence data generated in this study have been deposited in the DDBJ Sequence Read Archive under BioProject accession no. PRJDB39692. The individual BioSample accession nos. are SAMD01789812 for strain PS8, SAMD01789813 for R1, SAMD01789814 for R3, SAMD01789815 for R4, SAMD01789816 for R5, SAMD01789817 for R6, SAMD01789818 for R9 and SAMD01789819 for R10.

## Introduction

**Fig. 1.**
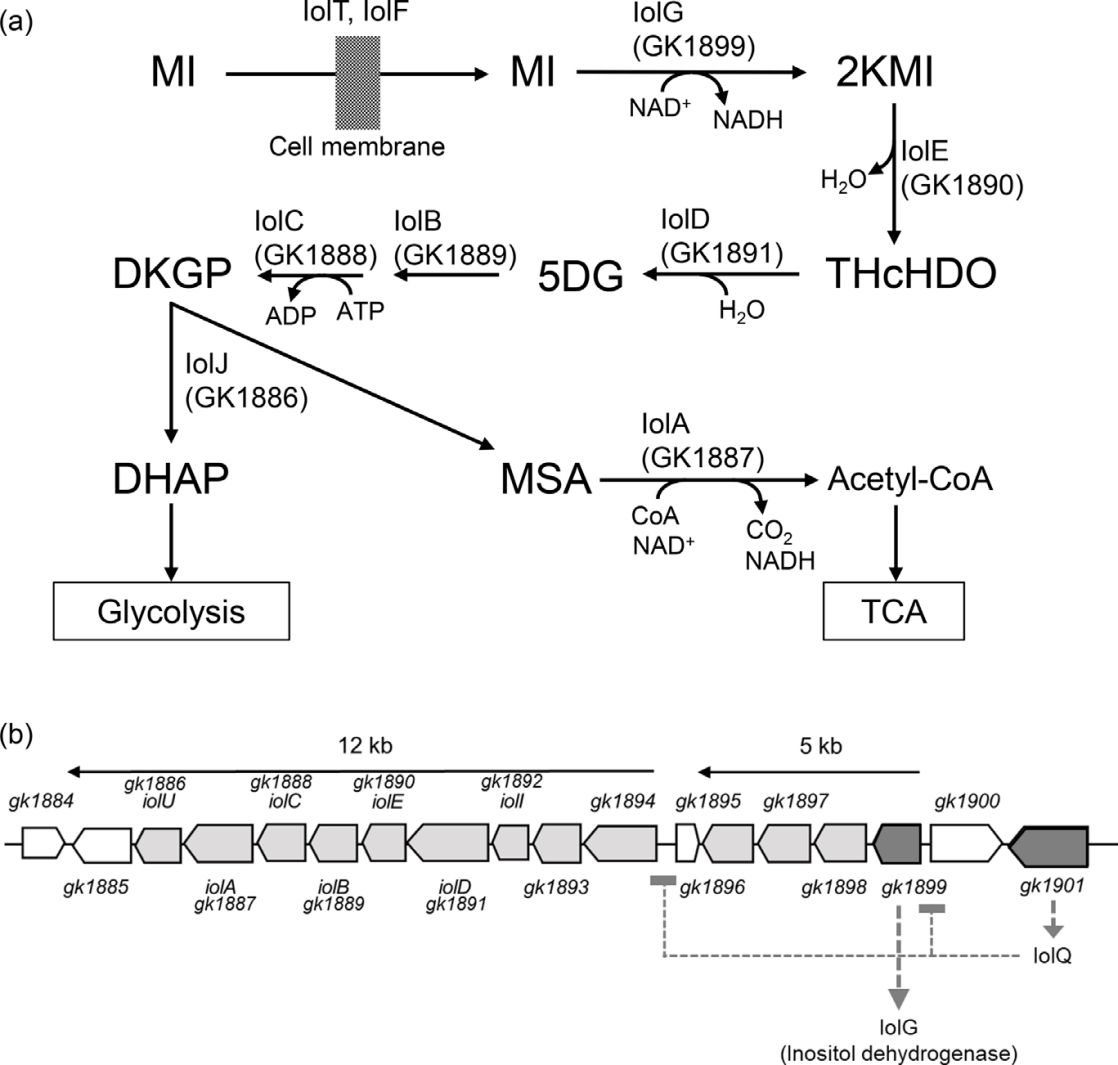
Inositol metabolism (a) and the *iol* gene organization (b) in *G. kaustophilus* HTA426. Enzymes of *B. subtilis* (the corresponding *G. kaustophilus* enzymes are the CDS products in parentheses) are shown associated with each reaction indicated by arrows. Compounds are shown by their abbreviations (see the main text).

Some bacteria, including *Bacillus subtilis*, a representative of Gram-positive bacteria, grow using *myo*-inositol (MI) as a carbon source. *B. subtilis* has been studied to elucidate its catabolism of MI involving the various *iol* genes (Fig. 1a). Most of the *iol* genes are encoded by a single transcriptional unit called the *iol* operon, which is regulated by the IolR repressor and induced in the presence of MI in the growth medium [1,2]. Each IolT and IolF transports MI into the cell [3], which is then oxidized to 2-keto-*myo*-inositol (2KMI) by inositol dehydrogenase, IolG, with the reduction of NAD^+^ into NADH [4]. IolE dehydrates 2KMI into 3D-(3,5/4)-trihydroxycyclohexane-1,2-dione (THcHDO) [5], followed by hydrolysis to 5-deoxy-d-glucuronic acid (5DG) by IolD [6]. 5DG is successively isomerized by IolB and phosphorylated by IolC into 2-deoxy-5-keto-d-gluconic acid 6-phosphate (DKGP), which acts as the inducer antagonizing the IolR repressor [2,6]. IolJ aldolase splits DKGP into dihydroxyacetone phosphate (DHAP), a glycolytic intermediate and malonic semialdehyde (MSA). Finally, IolA converts MSA into acetyl-CoA, releasing carbon dioxide and reducing NAD^+^ into NADH [6], and then, acetyl-CoA enters the TCA cycle.

*Geobacillus kaustophilus* HTA426 is a thermophilic Gram-positive bacterium that grows at higher temperatures from 42 to 74 °C, with an optimum at 60 °C [7]. HTA426 grows on MI as the sole carbon source, using a similar catabolic pathway to that of *B. subtilis* [8]. A complete set of *iol* genes for MI catabolism is split into two operons, respectively, of 5 kb and 12 kb in length [8] (Fig. 1b). These operons are both regulated by the binding of the IolQ repressor to their respective promoter regions [9]. In contrast to *B. subtilis*, MI itself inactivates IolQ to induce the expression of the *iol* operons [9].

**Fig. 2.**
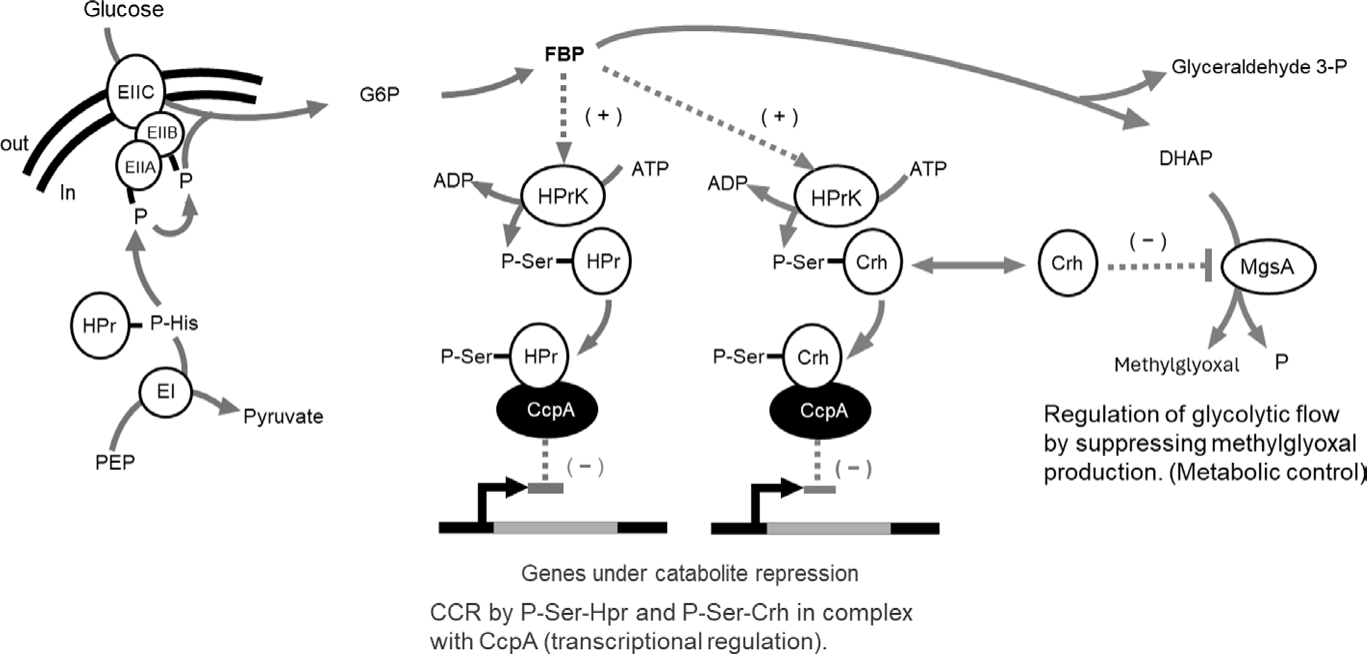
Schematic summary of the catabolite repression mechanism elucidated in *B. subtilis*.

In *B. subtilis*, the expression of the *iol* operon is regulated by carbon catabolite repression (CCR) [10], a global regulatory system in which the genes for the utilization of minor carbon metabolites are repressed when preferable carbon sources are available [11]. The mechanism of CCR in *B. subtilis*, in which glucose is the primary carbon source, has been elucidated (Fig. 2). Glucose is taken up into the cell via the phosphotransferase system (PTS), which is composed of enzyme I (EI), enzyme II (EII) and histidine-containing phosphocarrier protein HPr, encoded by *ptsH* [12]. In the presence of glucose, phosphoenolpyruvate (PEP) is generated via glycolysis, and EI transfers the phosphate group of PEP to the histidine 15 residue of HPr, which is then transferred to EII [13]. As soon as glucose passes through the plasma membrane into the cell, the phosphoryl residue on EII is transferred to glucose, generating glucose 6-phosphate (G6P), the initial product of glycolysis [14]. As G6P is catabolized through the glycolytic pathway, intracellular levels of fructose-1,6-bisphosphate (FBP) and ATP increase [14,15]. When intracellular levels of FBP accumulate, the HPr kinase/phosphatase, HPrK, is activated, leading to the phosphorylation of the serine 46 residue of HPr to yield P-Ser-HPr. P-Ser-HPr forms a complex with the catabolite control protein A (CcpA) that binds to nucleotide sequences containing the *cre* element within the chromosome, thereby repressing transcription of various genes for secondary carbon source metabolism [14,15].

There is a paralog of HPr, known as the carbon-flux-regulating HPr (Crh), which is also involved in CCR by a similar mechanism. Like HPr, Crh is also phosphorylated to P-Ser-Crh by HPrK [16]. It was also reported that Crh interferes with glycolysis by inhibiting MgsA [17] (Fig. 2). MgsA converts DHAP to methylglyoxal and initiates a glycolytic bypass to prevent the accumulation of phosphorylated sugars. However, methylglyoxal is toxic, and its production must be appropriately controlled. Under conditions where FBP concentration is low and HPr kinase activity is reduced, unphosphorylated Crh interacts with and inhibits MgsA. Thus, Crh controls the flux of glycolysis, but this function is lost when Crh is phosphorylated by HprK [17].

In *G. kaustophilus* HTA426, the genes encoding the counterpart of the *B. subtilis* CCR system, including GK3082 (HprK), GK0995 (HPr), GK2810 (CcpA), GK3063 (Crh) and GK2184 (MgsA), are present [7]. Moreover, the consensus sequence for IolQ binding site [9] bears a resemblance to that of the *cre* element for CcpA binding in *B. subtilis*. Therefore, it was predicted that CCR might function in the regulation of *iol* operons in HTA426. However, since the *iol* operons of HTA426 are unaffected in the presence of glucose [9], the CCR system in HTA42 may be similar yet distinct from that of *B. subtilis*. In a previous study, we conducted random mutagenesis on HTA426 in the presence of ethyl methanesulphonate (EMS) to isolate the mutant strain PS8 that does not grow on MI as the sole carbon source [8]. It was found that the *iol* operons of PS8 were constitutively repressed, even in the presence of MI [8]. Therefore, this study aimed to investigate why the *iol* operons are not induced in PS8. The results led to the discovery of an atypical CCR of the *iol* operons in *G. kaustophilus* HTA426 that occurs in the presence of ribose rather than glucose.

## Methods

### Bacterial strains, plasmids and oligonucleotide primers

Bacterial strains and plasmids used in this study are listed in Table 1. Strains of *G. kaustophilus* and *B. subtilis* were maintained in LB medium [18]. Strains of *G. kaustophilus* and *B. subtilis* grew at 60 °C and 37 °C, respectively, with shaking at 200 r.p.m. for liquid cultures. When needed, the medium was supplemented with antibiotics, including 25 mg l^−1^ of ampicillin, 25 mg l^−1^ of bleomycin, 10 mg l^−1^ of chloramphenicol, 1 mg l^−1^ of erythromycin, 100 mg l^−1^ of hygromycin, 10 mg l^−1^ of kanamycin, 100 mg l^−1^ of spectinomycin and 25 mg l^−1^ of tetracycline. One milligram per litre of uracil was also added as needed. For the growth test and mutant screening of *G. kaustophilus*, minimal medium was used [8], which contained 25 mM of C6 carbon sources or 30 mM of C5 carbon sources. For the *iol* induction test of *G. kaustophilus*, minimal medium containing 0.5% casamino acids was used, which was supplemented with and without 10 mM of C6 carbon sources and 12 mM of C5 carbon sources. For the *iol* induction test of *B. subtilis*, S6 medium [1] containing 0.5% casamino acids was used, which was supplemented with and without 10 mM of C6 carbon sources and 12 mM of C5 carbon sources. Oligonucleotide primers used in this study are listed in Table 2.

**Table 1.** Bacterial strains and plasmids used in this study

Strain and plasmid	Derivation and relevant genotype	Source or reference
***G. kaustophilus***		
HTA426	Wild-type strain	[7]
PS8	A mutant of HTA426, *iol* defective	[8]
R1	A suppressor mutant of PS8	This work
R3	A suppressor mutant of PS8	This work
R4	A suppressor mutant of PS8	This work
R5	A suppressor mutant of PS8	This work
R6	A suppressor mutant of PS8	This work
R9	A suppressor mutant of PS8	This work
R10	A suppressor mutant of PS8	This work
MK72	A mutant of HTA426, Δ *pyrF* Δ*pyrR*	[19]
YS202	A mutant of MK72, Δ*gk1901* (*iolQ*)::*kan*	[9]
Δrbs	A mutant of MK72, Δ*gk3226-gk3230*	This work
TKG003	A mutant of HTA426, *Δgk3082* (*hprK*)::*hph5*	This work
TKG004	A mutant of PS8, *Δgk3082* [*hprK(G268R*)]::*hph5*	This work
TKG005	A mutant of HTA426, *Δgk2810* (*ccpA*)::*hph5*	This work
TKG006	A mutant of PS8, Δ*gk2810* (*ccpA*)::*hph5*	This work
***B. subtilis***		
168	*trpC2*	Laboratory stock
YNB211	*trpC2 amyE*::(P*spank-rap_pLS20_ spc*) *epr*::(*PrpsO-dam ble*) *yhfK*::(*oriT_pLS20_ erm*), *harbouring* pLS20catΔoriT (*cat*)	[20]
RK03	*ΔyncM-yndN gerA*::*comK comS*(*hyg*) *ΔnucA*(*ble*)	Laboratory stock
TKB005	*trpC2 amyE*::(*Pspank-rap_pLS20_ spc*) *epr*::(*PrpsO-dam ble*) *yhfK*::(*oriT_pLS20_ erm*) *aprE*::[*gk3082* (*hprK*)::*hph5*], harbouring pLS20catΔoriT (*cat*)	This work
TKB006	*trpC2 amyE*::(P*spank-rap_pLS20_ spc*) *epr*::(P*rpsO-dam ble*) *yhfK*::(*oriT_pLS20_ erm*) *aprE*::[*gk2810* (*ccpA*)::*hph5*] pLS20catΔoriT (*cat*)	This work
***E. coli***		
BR408	F^-^*proA +B+lacI^q^* Δ(l*acZ*)M15 *zzf*::Tn10/*fhuA2 glnV* Δ(*lac-proAB*) *thi*-1 Δ(*hsdS-mcrB*)5 Δ*dcm::lacZ, dam^+^,* harbouring pUB307 (plasmid for conjugation, *tet*) and pIR408 (plasmid for gene methylation, *cat*)	[19]
**Plasmids**		
pUCG18T	*kan amp*	[19]
pUCG18T-hprK	pUCG18T derivative, P*_sigA_-gk3082* (*hprK*)	This work
pUCG18T-hprK(G268R)	pUCG18T derivative, P*_sigA_- gk3082* [*hprK(G268R*)]	This work
pUCG18T-crh	pUCG18T derivative, P*_sigA_-gk3063* (*crh*)	This work
pGKE25	A pUC19 derivative, *oriT_pUB307_ amp pyrF*	[19]
pGKE25-rbs-deletion	pGKE25 carrying the Δ*gk3226-gk3230* fragment	This work

**Table 2.** Oligonucleotide primers used in this study

Name	Sequence (5′→3′)*
Hind3-sigA-F	GTAAAACGACGGCCAGTGCCAAGCTTGCATGCCTGCAGCTTCGCCTCATCCGCACGATTTC
sigA-R-3082	GGATCACGCCCCCGTTACGACCGGTTGCGGTATCGTATTTATTCTCGGCAGC
gk3063-F	GCTGCCGAGAATAAATACGATACAGCCGGTCGATTTCCATAAATCGC
gk3063-R	GGGGATCCTCTAGAGTCGACCTGCAAAAAAAGGCTGCTTCCGACAAAAAC
Pst1-sigA-F	CCAGTGCCAAGCTTGCATGCCTGCACTTCGCCTCATCCGCACGATTTC
sigA-R-3063	GCGATTTATGGAAATCGACCGGCTGTATCGTATTTATTCTCGGCAGC
gk3082-F-sigA	TCGGCTTGCTGCCGAGAATAAATACGATACCGCAACCGGTCGTAACGGGGGCGTG
gk3082-R-Xba1	TACCCGGGGATCCTCTAGATGATAACGGCTGCCCTAGGAAAACAGGGCAGCC
pGKE-rbs-UF	TTCGAGCTCGGTACCCGGGGATCCTCTAGAGGCGGATACATCATCAAAATCAAGC
rbs-UR	CAGTCGACGTCAAGGAGGGGATTGACTTGACGCGTAATATTCTTTCGCGTGAACG
rbs-DF	TCAAGTCAATCCCCTCCTTGACGTC
rbs-DR-pGKE	AACAGCTATGACCATGATTACGCCAAGCTTAACGCGGCGTATAATAGAACAAAGC
aprE-1f	CCCCCTCCGCAAAACGCGGATCATTGG
aprE-1r	CTCTCGCTATTTCCGTAGAGACTCG
aprE-2f	GCTGCACAATAATAGTAAAAAGAAGCAGG
aprE-2r	CGCTGATTACAACATTGGTGACGCTGCC
hprK-nF-f	TTATTTCGAGTCTCTACGGAAATAGCGAGAGCCTTCAATCCAGAAACGGCCAACCGAATAC
hprK-nF-r	CTTCATCATCGGTCATAAAATCCGTATCCTTCTTTGCACCGACATTACGCCAGGGATCATC
hprK-nR-f	GATGTGCTGCAAGGCGATTAAGTTGGGTAACGCGTCAACGTTTCATAAAACGACAGCTCCG
hprK-nR-r	CCTGCTTCTTTTTACTATTATTGTGCAGCCGTTACGATCCTCTTGTTCATTGCGTTGG
hph5F	AAGGATACGGATTTTATGACCGATGATGAAGTCAGCAATTTGCGGAAATAACTTGCAACGC
hph5R	GTTACCCAACTTAATCGCCTTGCAGCACATCGAATTCACTGGCCGTCGTTTTACAACGTCG
ccpA-nF-f	TTATTTCGAGTCTCTACGGAAATAGCGAGAGCGACGATCCGAACGTTTGCACGTTTTCCATTC
ccpA-nF-r	CTTCATCATCGGTCATAAAATCCGTATCCTTGCGTTTGAAGCCGTGACGTATTTGCTGGA
ccpA-nR-f	GATGTGCTGCAAGGCGATTAAGTTGGGTAACCTGCTCATAGTCGATGTTGACCGAGGGAATC
ccpA-nR-r	CCTGCTTCTTTTTACTATTATTGTGCAGCGATTTGGCGGTCATCAGTGAAATCGTCACC
gk1894RT-F	TTTTCGTCAACCCTTGAACC
gk1894RT-R	GAGGGATGGCAAGTACATCG
gk1899RT-F	ATTCCTCGAGGCTTTTGACC
gk1899RT-R	TGACTCGGGTGAAAGTAGGG
gk3063RT-F	CTTTTCGATCGCTTCTTGC
gk3063RT-R	AGAAGCGAACCGTTTTTCC
gk0995RT-F	TTGAGTACAACGGCAAAACG
gk0995RT-R	CTTCCTTTGCCAACGTATCG
gk0103RT-F	CATGACTGACCCGTATGTCG
gk0103RT-R	AATTTCTTGACGGTGGTTCG
ptsHRT-F	TAGCAAATACGACGCTGACG
ptsHRT-R	CGTTAAGAGCATCGTTTTCG
crhRT-F	GGTAACCTCAGTGCCTGTGC
crhRT-R	TTCAACAGAAAGTGGAAGTTCG
fusART-F	ATGGAGCAGGAACAAGAACG
fusART-R	GCGTCAAGTACAGCAACAGC

*Restriction enzyme sites are underlined.

### Construction of bacterial strains

Plasmids pUCG18T-crh, pUCG18T-hprK and pUCG18T-hprK(G268R) (Table 1) were constructed as follows:

Genomic DNA from HTA426 was used as a template to PCR amplify the *sigA* promoter, using primer pair Pst1-sigA-F and sigA-R-3063 (Table 2). The intact *gk3063* (*crh*) coding region, including the ribosome-binding site, was amplified using the primer pair gk3063-F and gk3063-R (Table 2). The resulting PCR fragments were ligated by recombinant PCR using the primer pair Pst1-sigA-F and gk3063-R. This PCR fragment was trimmed with *Pst*I and *Xba*I and ligated with the arms of similarly digested pUCG18T, to generate pUCG18T-crh (Table 1). A second DNA fragment containing the *sigA* promoter region was amplified from HTA426 DNA using the primer pair Hind3-sigA-F and sigA-R-3082 (Table 2). The fragment containing the *gk3082* (*hprK*) coding region, including the ribosome-binding site, was amplified from HTA426 DNA using the primer pair gk3082-F-sigA and gk3082-R-Xba1 (Table 2). The fragment containing the *gk3082* [*hprK*(G268R)] coding region was also amplified from PS8 DNA using the same primer pair. The *sigA* promoter fragment and the *hprK* fragment or *hprK*(G268R) fragment were ligated by recombinant PCR using the primer pair Hind3-sigA-F and gk3082-R-Xba1. These recombinant PCR fragments were each trimmed with *Hind*III and *Xba*I and inserted into pUCG18T, yielding pUCG18T-hprK and pUCG18T-hprK(G268R) (Table 1). After confirming their correct construction by DNA sequencing, each of the plasmids was transformed into *Escherichia coli* strain BR408, and from there into *G. kaustophilus* strains HTA426, PS8 or the suppressor mutants via conjugative transfer [19].

The mutant strain Δrbs of *G. kaustophilus* (Table 1) was created from strain MK72 (Table 1), an uracil auxotrophic mutant of HTA426, as follows:

First, using HTA426 DNA as a template, the upstream region (~1.5 kb long) of *gk3226* was amplified with the primer pair pGKE-rbs-UF and rbs-UR (Table 2), and the downstream region (~1.5 kb long) of *gk3230* was amplified using primer pairs rbs-DF and rbs-DR-pGKE (Table 2). These fragments were ligated via recombinant PCR using the primer pairs pGKE-rbs-UF and rbs-DR-pGKE. The resulting fragment was ligated with the arms of *Hind*III- and *Xba*I-digested pGKE25 using NEBuilder HiFi DNA Assembly Master Mix (NEB), yielding plasmid pGKE25-rbs-deletion (Table 1). This plasmid was transformed into *E. coli* BR408 and from there into *G. kaustophilus* strain MK72 (Table 1) via conjugation [19]. As pGKE25-rbs-deletion does not replicate in *G. kaustophilus*, selection was made for an integrant at the upstream or downstream of the *gk3226-gk3230* region of the chromosome via a single-crossover recombination. The resulting integrant was prototrophic for uracil, acquiring *pyrF* from pGKE25-rbs-deletion, and formed colonies on minimal medium containing 0.5% casamino acids but not uracil. Next, the region of the chromosome corresponding to the inserted region on plasmid pGKE25-rbs-deletion was deleted via an intrachromosomal recombination event. The prototrophic strain was inoculated into minimal medium containing 0.5% casamino acids supplemented with both 1 mg l⁻¹ of uracil and 50 mg l⁻¹ of 5-fluoroorotic acid (5-FOA), selecting for colonies exhibiting both 5-FOA resistance and uracil auxotrophy at 60 °C. The 5-FOA-resistant and uracil auxotrophic *G. kaustophilus* colonies correspond to variants in which the region corresponding to plasmid pGKE25-rbs-deletion is lost via intrachromosomal recombination. However, approximately half of these regained the original genetic context of strain MK72, while the remainder had the *gk3226-gk3230* region deleted. One such variant, with the *gk3226-gk3230* deletion, was designated as strain Δrbs.

The mutant strains TKG003, TKG005, TKG004 and TKG006 of *G. kaustophilus* (Table 1) were created as follows:

For the creation of TKG003 and TKG004, the upstream and downstream regions (each ~1.0 kb long) of the *aprE* gene in *B. subtilis* 168 were first amplified by PCR using the primer pairs aprE-1f and aprE-1r and aprE-2f and aprE-2r (Table 2) to obtain Fragments 1 and 2, respectively. Subsequently, from the upstream and downstream regions (each ~1.0 kb long) of *gk3082* (*hprK*) were PCR amplified from HTA426 DNA using the primer pairs hprK-nF-f and hprK-nF-r and hprK-nR-f and hprK-nR-r (Table 2), yielding Fragments 3 and 4, respectively. Furthermore, the fragment containing the hygromycin resistance gene (*hph5*) was amplified from *B. subtilis* strain RK03 DNA by PCR using the primer pairs hph5F and hph5R (Table 2), yielding Fragment 5. These five fragments were ligated in the order of Fragments 1, 3, 5, 4 and 2 using the NEBuilder HiFi DNA Assembly Master Mix and amplified by PCR using the primer pair aprE-1f and aprE-2r. This PCR fragment was transformed into the *B. subtilis* strain YNB211 (Table 1), selecting for hygromycin resistance. The resulting strain, TKB005, had a copy of the generated PCR fragment integrated into the chromosome at the *amyE* locus via a double-crossover recombination. By conjugating TKB005 with HTA426 and PS8, mediated by the conjugative plasmid pLS20 [20,21], hygromycin-resistant transconjugants TKG003 and TKG004 were obtained, in which *hph5* replaced *gk3082* (*hprK*) and *gk3082* [*hprK*(G268R)], respectively.

For the generation of TKG005 and TKG006, Fragments 6 and 7, corresponding to the upstream and downstream regions (each ~1.0 kb long) of *gk2810* (*ccpA*), were amplified from HTA426 DNA by PCR using the primer pairs ccpA-nF-f and ccpA-nF-r and ccpA-nR-f and ccpA-nR-r (Table 2), respectively. The resulting PCR fragments were ligated in the order of Fragments 1, 6, 5, 7 and 2, and amplified as described above. The resulting PCR fragment was transformed into YNB211 to obtain another hygromycin-resistant *B. subtilis* strain TKB006 (Table 1). By conjugating TKB006 with HTA426 and PS8, as described above, the hygromycin-resistant strains TKG005 and TKG006 were obtained, in which *gk2810* (*ccpA*) was replaced by *hph5*.

### Screening of the base substitutions in mutant strains of *G. kaustophilus*

Draft genome sequencing of *G. kaustophilus* mutant strains was performed using the Genome Analyzer IIx, following the manufacturer’s protocols, with generation of 75- or 100-bp paired-end reads connected to 6-bp index tags. The purpose of this sequencing was not to determine the complete genome sequence of each mutant strain, but to perform comparative analysis to identify differences with the parental strain HTA426. The pair-end reads were aligned to the HTA426 genome (GenBank accession number: GCA_000009785.1) using BWA software (ver. 0.5.1) with default parameters. Possible variations (SNP/Indel) were listed using SAMtools software (ver. 0.1.9). The raw sequence data generated in this study have been deposited in the DDBJ Sequence Read Archive under BioProject accession number PRJDB39692. The individual BioSample accession numbers are SAMD01789812 for strain PS8, SAMD01789813 for R1, SAMD01789814 for R3, SAMD01789815 for R4, SAMD01789816 for R5, SAMD01789817 for R6, SAMD01789818 for R9 and SAMD01789819 for R10.

### Inositol dehydrogenase enzyme assay

Inositol dehydrogenase activity was measured as described previously [1,4, 8]. Bacterial cells (three OD_600_ units) were harvested by centrifugation and resuspended in 0.1 ml of 100 mM Tris-HCl (pH 8.0). After the addition of 5 µl of 100 mM Tris-HCl (pH 8.0) containing 10 mg ml^−1^ lysozyme, the mixture was incubated at 37 °C for 30 min. Fifty microlitre aliquots were subjected to ultrasonication for 5 min at 4 °C using a BIORUPTOR (Cosmo Bio, UCD-250). The samples were centrifuged at 20,380 ***g*** for 15 min at 4 °C. The supernatant was used to measure inositol dehydrogenase activity at 55 °C for *G. kaustophilus* and 37 °C for *B. subtilis*. The increase in absorbance at 340 nm was recorded in the presence of 0.5 mM *β*-NAD^+^ and 50 mM MI in 0.1 M Tris-HCl (pH 8.0). The activity, NADH (nmols) produced min^−1^ mg protein^−1^, was calculated based on the molar coefficient of NADH as 6.22×10^3^ M^−1^ cm^−1^. Data obtained from three independent replicate experiments were subjected to statistical analysis using Student’s t-test to determine the presence of significant differences.

### Transcription analysis (reverse transcription quantitative PCR)

RNA extraction from bacterial cells and reverse transcription quantitative PCR were performed as follows.

Bacterial cells (12 OD_600_ units) were harvested by centrifugation. The cells were suspended in 640 µl LETS buffer (100 mM LiCl, 10 mM EDTA, 10 mM Tris-HCl, pH 7.5, 1% SDS), to which 0.4 ml of glass beads (φ0.5 mm) and 0.4 ml of phenol/chloroform/isoamyl alcohol (25/24/1) were added. The cells were disrupted by vigorous shaking using Beads Crusher µT-12 (TAITEC) at 3,000 r.p.m. for 1 min, followed by centrifugation at 20,380 ***g*** for 5 min at room temperature. The supernatant (0.55 ml) was mixed with an equal volume of phenol/chloroform/isoamyl alcohol (25/24/1) and centrifuged at 20,380×*g* for 5 min at room temperature. 0.4 ml of the upper water layer was mixed with 40 µl of 1 M LiCl and 1 ml of 100% ethanol, incubated for 1 h at −20 °C, and then centrifuged at 20,380 ***g*** for 20 min at 4 °C. The resulting pellet was washed with 70% ethanol, dried and resuspended in 0.1 ml of RNase-free water. Then, RNA was further purified using the RNeasy Mini Kit (QIAGEN).

The purified RNA samples were converted into cDNA using RT-PCR Quick Master Mix (TOYOBO) and subjected to quantification of transcription levels of the target genes using THUNDERBIRD^®^ SYBR^®^ qPCR Mix (TOYOBO) with the specific primer pairs as follows (Table 2): for *G. kaustophilus gk1894*, gk1984RT-F and gk1984RT-R; for *gk1899* (*iolG*), gk1899RT-F and gk1899RT-R; for *gk3063* (*crh*), gk3063RT-F and gk3062RT-R; for *gk0995* (*ptsH*), gk0995RT-F and gk0995RT-R; for *gk0103*, gk0103RT-F and gk0103RT-R; for *B. subtilis ptsH*, pstHRT-F and ptsHRT-R; for *crh*, crhRT-F and crhRT-R; and for *fusA*, fusART-F and fusART-R. The transcription level of each gene was calculated using the 2^-ΔΔ*C*^_T_ method [22]. For *G. kaustophilus*, *gk0103*, which encodes translation elongation factor G, was used as the control considered to have a constant expression level [23]. For *B. subtilis*, *fusA* was used instead [24]. Data obtained from three independent replicate experiments were subjected to statistical analysis using Student’s t-test to determine significance.

## Results

### Suppressor mutants of *G. kaustophilus* PS8 that regained *iol* induction

Mutant strain PS8 of *G. kaustophilus*, obtained previously via EMS mutagenesis [8], constitutively represses the expression of the *iol* genes. Consequently, PS8 can scarcely grow using MI as the sole carbon source. PS8 was inoculated into liquid minimal medium containing MI as the sole carbon source for several days to select for spontaneous suppressor mutants. The culture was plated onto minimal agar medium with MI as the carbon source, and liquid minimal medium containing MI was inoculated with several large colonies. Seven isolates exhibiting restored growth on MI similar to that of the parental strain HTA426 were selected and designated as the suppressor mutants R1, R3, R4, R5, R6, R9 and R10.

**Fig. 3.**
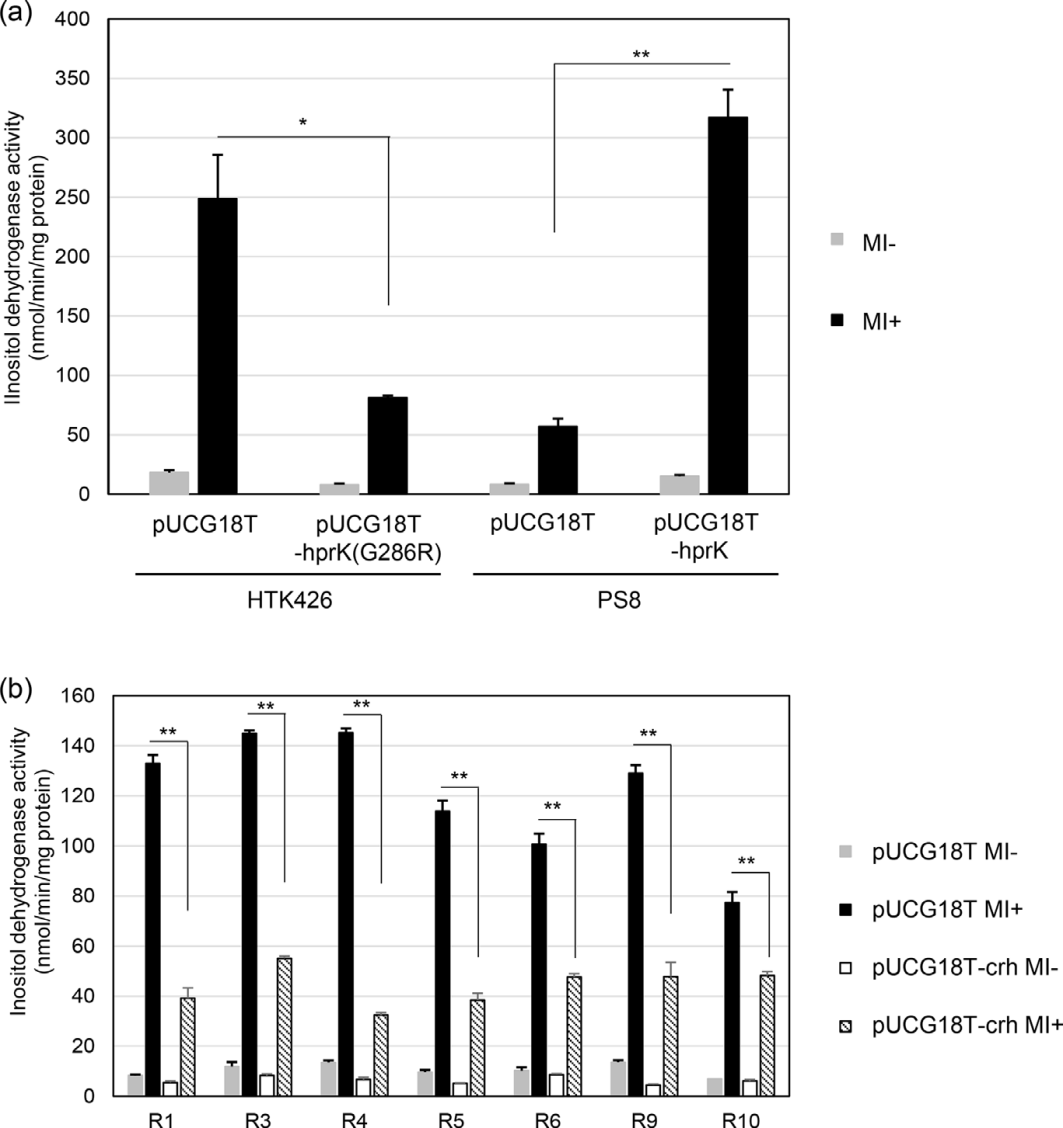
Inositol dehydrogenase activities in HTA426 and PS8 (a) and in the suppressor mutant strains of PS8 (b). The mean±sd of three independent measurements is shown.

A significant level of inositol dehydrogenase activity was observed in the parental strain HTA426, in which the empty vector pUCG18T has been introduced in relation to the subsequent experiment, grown on MI (Fig. 3a), due to the expression of the IolG enzyme encoded by *gk1899* (Fig. 1). However, this induction was lost in PS8 (Fig. 3a). In all seven suppressor mutants, the inositol dehydrogenase activity was significantly induced in the presence of MI, albeit to a lower level than the HTA426 (Fig. 3b). It was, therefore, of interest to understand the nature of the mutations in the suppressor mutants and how they led to the restoration of *iolG* induction.

### Mutations in PS8 and its suppressor mutants

To identify the mutations in PS8 and its suppressor mutants, we generated draft genome sequences. These sequences for each strain covered more than 99% of the HTA426 genome, with overlapping depths ranging from 27 to 44. Ambiguous differences, including competing substitutions, insertions and deletions detected among the individual reads, were excluded.

**Fig. 4.**
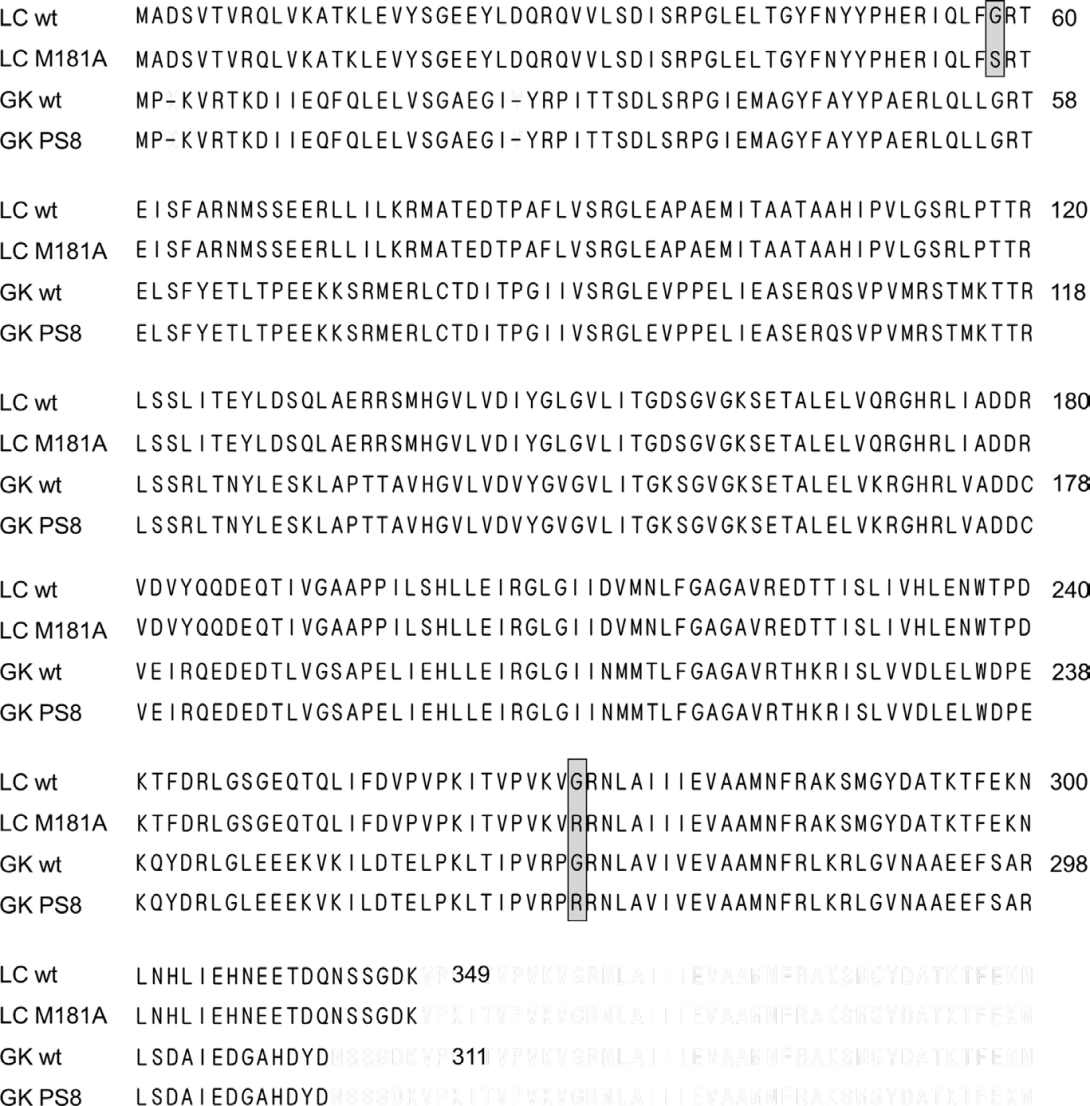
Alignment of amino acid sequences of *hprK* of the parental strain (LC wt) and its mutant M181A (LC M181A of *L. casei* and strain HTA426 (GK wt) and its mutant PS8 (GK PS8) of *G. kaustophilus*. Amino acid substitutions are indicated by grey shading with borders.

We identified at least 19 significant mutations in PS8 (Table 3). Among these, the missense mutation substituting glycine 268 for arginine (G268R; GGG to AGG) in *gk3082* (*hprK*) was common in PS8 as well as all seven suppressor mutants, and the mutated gene was designated as the *hprK*(G268R) allele. This gene is presumed to encode HPr kinase/phosphorylase, which is involved in CCR in various Gram-positive bacteria. HPr kinase/phosphorylase is bifunctional in Gram-positive bacteria; it exerts the kinase activity to generate P-Ser-HPr and P-Ser-Crh in response to increased intracellular FBP concentration and possesses phosphorylase activity that dephosphorylates P-Ser-HPr and P-Ser-Crh to their non-phosphorylated form under conditions where catabolite repression does not occur [25]. In *Lactobacillus casei* strain M181A, a double mutation of glycine 58 to serine (G58S) and glycine 270 to arginine (G270R) in *hprK* resulted in the accumulation of P-Ser-HPr (and probably also P-Ser-Crh), due to impaired phosphorylase activity; however, it remained to be confirmed whether either or both of these mutations were involved in this [26]. The alignment of the amino acid sequences of *L. casei hprK* and *G. kaustophilus hprK* indicates that G270R in the former corresponds to G268R in the latter (Fig. 4).

**Table 3.** Mutations in the strain PS8 and its suppressor mutants of *G. kaustophilus*

Position	Gene	Description	Mutant strain*							
			PS8	R1	R5	R6	R10	R3	R4	R9
32631	*gkt003*	tRNA-Ile	70>+G	70>+G	70>+G	70>+G	70>+G	70>+G	70>+G	
112031	*gk0074*	Lysyl-tRNA synthetase, flank3	94>+G		94>+G		94>+G	94>+G		94>+G
695615	*gk0659*	ABC transporter permease, flank3		54>-C		54>-C			54>-C	
993503	*gk0969*	Hypothetical protein		#L219>+T		#L219>+T		#L219>+T	#L219>+T	#L219>+T
1004267	*gk0981*	Spore cortex-lytic enzyme					#R144>+C	#R144>+C		
1004270	*gk0981*	Spore cortex-lytic enzyme		#R143>+C			#R143>+C			#R143>+C
1004273	*gk0981*	Spore cortex-lytic enzyme		#R142>+C	#R142>+C	#R142>+C	#R142>+C		#R142>+C	#R142>+C
1034227	*gk1007*	Hypothetical protein, flank5		18>+A		18>+A		18>+A		18>+A
1045456	*gk1018*	Hypothetical protein	#S173>-A	#S173>-A		#S173>-A	#S173>-A		#S173>-A	#S173>-A
1149292	*gk1129*	Hypothetical protein	#A43>+G	#A43>+G	#A43>+G	#A43>+G		#A43>+G		
1221636	*gk1202*	50S ribosomal protein L19	#E58>-A	#E58>-A	#E58>-A	#E58>-A	#E58>-A		#E58>-A	#E58>-A
1221693	*gk1202*	50S ribosomal protein L19	#R77>-G						#R77>-G	
1221898		50S ribosomal protein L19, flank3	49>-T		49>-T		49>-T	49>-T	49>-T	49>-T
1319868	*gk1300*	Hypothetical protein	#R48>+C						#R48>+C	
1391177	*gk1369*	Hypothetical protein	#K169>+A		#K169>+A	#K169>+A			#K169>+A	
1391184	*gk1369*	Hypothetical protein	#R171>+G	#R171>+G			#R171>+G		#R171>+G	
1480252		Hypothetical protein, flank5						24>+A		24>+A
1497531	*gk1456*	Hypothetical protein	#Y101>+G		#Y101>+G	#Y101>+G			#Y101>+G	
1614698	*gk1583*	Hypothetical protein		#A224V	#A224V	#A224V	#A224V	#A224V	#A224V	#A224V
1836688	*gk1811*	Hypothetical protein						#D147>+G	#D147>+G	
1836715	*gk1811*	Hypothetical protein	#R138>+G			#R138>+G	#R138>+G			
2235808	*gk2194*	Hypothetical protein	#Q176>-C				#Q176>-C			#Q176>-C
2282831		Hypothetical protein, flank3	38>+T	38>+T	38>+T			38>+T		38>+T
2356064	*gk2331*	Pyrroline-5-carboxylate reductase	#R32>+C		#R32>+C		#R32>+C	#R32>+C		
2526915	*gk2507*	Coproporphyrinogen III oxidase								#L71L
2706176		Transcriptional regulator NrdR, flank5	94>+A		94>+A		94>+A	94>+A	94>+A	
2779831	*gk2756*	Argininosuccinate lyase	#R123Q							
2941861	*gkt084*	tRNA-Ser	60>+G	60>+G			60>+G			
**3084606**	***gk3063***	**Phosphocarrier protein Chr**		**#M1I**	**#M1I**	**#M1I**	**#M1I**			
**3084618**		**Phosphocarrier protein Chr, flank3**						**5C>T**	**5C>T**	**5C>T**
**3101997**	***gk3082***	**HPr kinase/phosphorylase**	**#G268R**	**#G268R**	**#G268R**	**#G268R**	**#G268R**	**#G268R**	**#G268R**	**#G268R**
3439359	*gk3398*	Iron-sulphur oxidoreductase		#Q693K	#Q693K			#Q693K		#Q693K
3439360	*gk3398*	Iron-sulphur oxidoreductase			#D692E			#D692E		#D692E

*Notation for mutation sites (examples of notation):

#A224V: an amino acid substitution at codon 224 of the CDS from A to V.

#L219>+T: an insertion of T at codon 219 of the CDS.

#S173>-A: a deletion of A at codon 173 of the CDS.

5C>T: a nucleotide substitution in the intergenic region at nucleotide position 5 from the start or end of the CDS from C to T.

70>+G: an insertion of G in the intergenic region at nucleotide position 70 from the start or end of the CDS.

54>-C: a deletion of C in the intergenic region at nucleotide position 54 from the start or end of the CDS.

Mutations occurring in *gk3063* (*crh*) and *gk3082* (*hprK*) are indicated in bold type.

Analysis of mutations specific to the suppressor strains revealed two distinct types of mutation in *gk3063* (*crh*), which is presumed to encode the Crh protein. In the suppressor strains R1, R5, R6 and R10, the start codon was altered from methionine to isoleucine (M1I; AUG to AUA). In the case of suppressor strains R3, R4 and R9, base substitutions were detected in the region of the presumptive ribosome-binding site (flank3, 5C>T; GGAGATG to GGAGATA) 5 bp upstream of the start codon on the coding strand. Both types of mutation suggest potential defects in *crh* translation. In *gk1583*, which encodes a hypothetical conserved protein, a missense mutation of the 224th alanine residue to valine (A224V; GCG to GUG) was commonly observed across all suppressor mutants. However, this mutation was not investigated further. Although we anticipated that only a small number of mutations would arise in the suppressor mutants, additional mutations were observed sporadically, and their total number exceeded this expectation (Table 3); the reason for this remains unclear.

### The *hprK*(G268R) allele caused constitutive *iol* repression in PS8, and defects in translation of *crh* restored inducible repression in the suppressor mutants

Based on the results of mutation screening, we formulated the following hypothesis: in PS8, the *hprK*(G268R) allele leads to the accumulation of P-Ser-Crh, which represses the expression of the *iol* genes. Conversely, in the suppressor mutants, the failure to translate *crh* prevents the accumulation of P-Ser-Crh and its associated repression.

To test this hypothesis, we introduced an additional plasmid-encoded copy of *hprK*(G268R) into HTA426 and observed a significant reduction of inositol dehydrogenase activity in the presence of MI (Fig. 3a). Additionally, when the wild-type copy of *gk3082* (*hprK*) was introduced into PS8 on another plasmid, inositol dehydrogenase activity became inducible by MI. These results support our hypothesis that the *hprK*(G268R) is the cause of the failure to induce the *iol* genes in PS8.

To provide additional evidence that confirms that suppression was due to the *crh* mutations, a plasmid-encoded copy of *gk3063* (*crh*) was introduced into each of the suppressor mutants (Fig. 3b). The inositol dehydrogenase activities of the suppressor mutants with the plasmid-encoded copy of *gk3063* (*crh*) in response to MI were significantly reduced compared to those with an empty plasmid. These results provide additional support for our hypothesis that the suppressor mutants had a reduced amount of Crh because their mutations caused defects in the translation of *gk3063* (*crh*), leading to the restoration of the induction of the *iol* genes.

### Ribose represses the *iol* operons

The data above indicated that the *iol* genes of *G. kaustophilus* could potentially be regulated by a CCR-like mechanism involving HPrK and Crh. However, the precise molecular mechanism and the physiological significance of this regulation are currently unclear. To better understand the system, we analysed the effects of various carbon sources on the CCR in strain HTA426. Initially, we determined the mean generation time (*T*_d_) during growth on various carbon sources. Interestingly, the *T*_d_ was shorter when HTA426 was cultured with C5 carbon sources (l-arabinose *T*_d_=0.77±0.02 h; d-xylose *T*_d_=0.75±0.03 h; d-ribose *T*_d_=0.73±0.02 h) than with C6 carbon sources (d-glucose *T*_d_=1.96±0.07 h; MI *T*_d_=1.30±0.04 h). Based on these results, we considered the possibility that HTA426 may prefer C5 carbon sources over C6 carbon sources. In *Clostridium acetobutylicum*, arabinose represses the expression of the xylose metabolism genes, and it was suggested that Crh may be involved in this repression [27]. Therefore, we postulated that in HTA426, CCR might be triggered by C5 carbon sources, involving Crh.

**Fig. 5.**
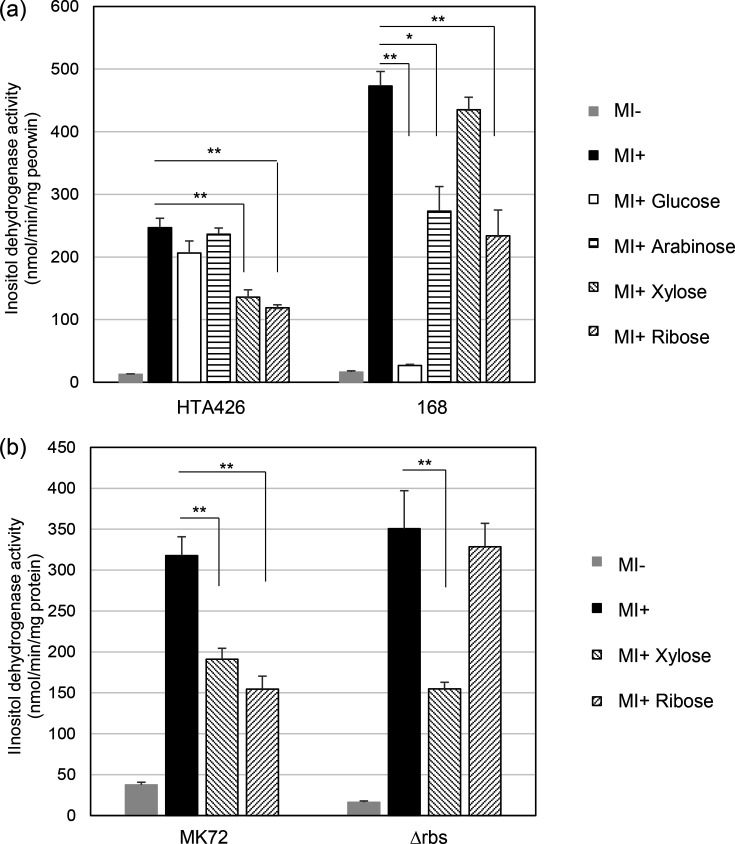
Inositol dehydrogenase activities in strains HTA426 and 168 (a) and MK71 and Δrbs (b) grown on various carbon sources. The mean±sd of three independent measurements is shown.

Considering the growth data, we investigated the impact of C5 carbon sources (e.g. arabinose, xylose and ribose) on the induction of inositol dehydrogenase by MI in strain HTA426. For comparison, we carried out parallel experiments on the closely related species, *B. subtilis* (Fig. 5a). In contrast to *B. subtilis*, neither glucose nor arabinose repressed inositol dehydrogenase activity in HTA426. However, xylose and, in particular, ribose exhibited significant repression. In *B. subtilis*, arabinose and ribose also showed repression, albeit at lower levels compared to the effect of glucose. The effects of xylose and arabinose differ between *B. subtilis* and *G. kaustophilus*, though the cause of this difference remains unclear. In any case, partial repression was observed in both *G. kaustophilus* and *B. subtilis* in response to the C5 carbon sources. Furthermore, while glucose acts as a potent inhibitory factor in *B. subtilis*, this is not the case in *G. kaustophilus*.

We then explored whether ribose-induced repression was a form of CCR by investigating whether disrupting ribose metabolism could alleviate this repression. We created a mutant strain Δrbs from strain MK72, the uracil auxotrophic mutant of HTA426 [19]. Δrbs lacks its *gk3226-gk3230* operon, which encodes the ribose transporter and the ribokinase genes. We confirmed that Δrbs was unable to grow when ribose was the sole carbon source (data not shown). Next, we determined the inositol dehydrogenase activity of this strain when grown on casamino acids with and without MI, ribose and xylose (Fig. 5b). While this enzyme was repressed by ribose in MK72, no repression by ribose was observed in Δrbs. The results suggest that an intermediate in ribose metabolism might be involved in the repression observed in the presence of ribose. On the other hand, xylose-induced repression still occurred. Therefore, xylose metabolism is independent of ribose metabolism, and the intermediate involved in CCR that arises from ribose metabolism may also be produced in xylose metabolism.

**Fig. 6.**
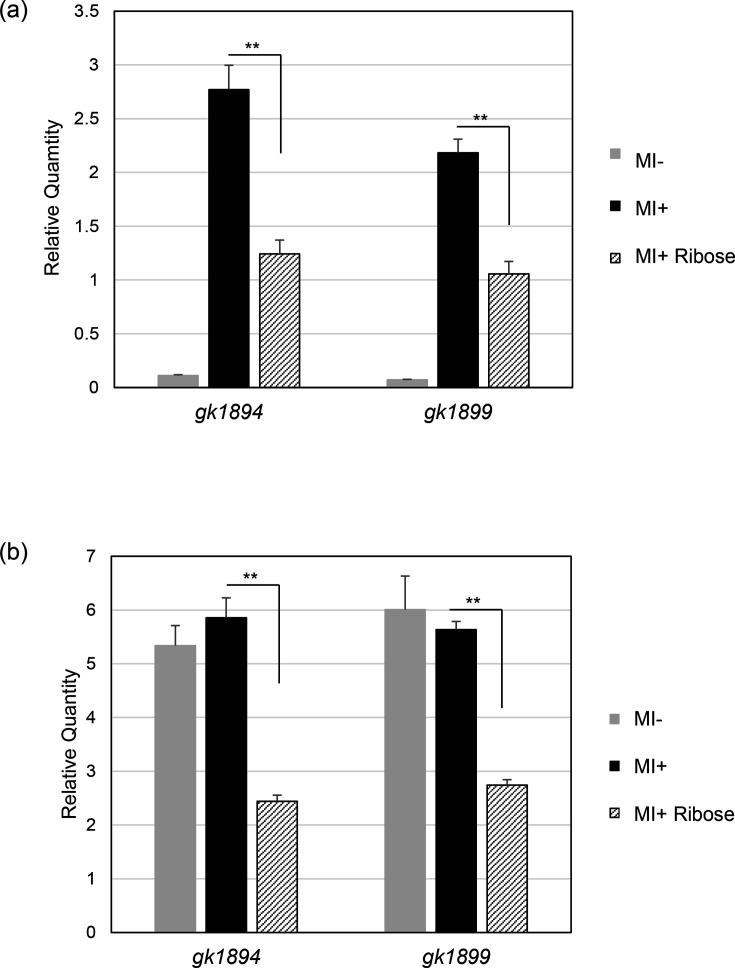
Transcription levels of *gk1894* (for the latter 12-kb operon) and *gk1899* (for the former 5-kb operon) in strains MK72 (a) and YS202 (b). The mean±sd of three independent measurements is shown.

The measurement of inositol dehydrogenase activity reflects the expression of the *iolG* gene encoded by *gk1899* and, therefore, the expression of only one of the two *iol* operons (Fig. 1b). To determine changes in transcription levels of both *iol* operons in response to ribose, mRNAs for the 5′ genes of both operons (*gk1894* and *gk1899*) were determined by reverse transcription quantitative PCR (Fig. 6a). The results indicate that the transcription of both *iol* operons was induced by MI and repressed significantly in the co-presence of ribose.

### Crh, not HPr, is primarily involved in ribose-induced catabolite repression of the *iol* operons in *G. kaustophilus*

To elucidate the mechanism of ribose-mediated repression of the *iol* operons, we first verified the effect of ribose on the *iol* transcription in the IolQ repressor-deficient strain YS202 (Fig. 6b). Since YS202 lacks IolQ [9], both the *iol* operons were transcribed even in the absence of MI. Furthermore, both operons were repressed by ribose, indicating that ribose-induced catabolite repression is independent of IolQ.

**Fig. 7.**
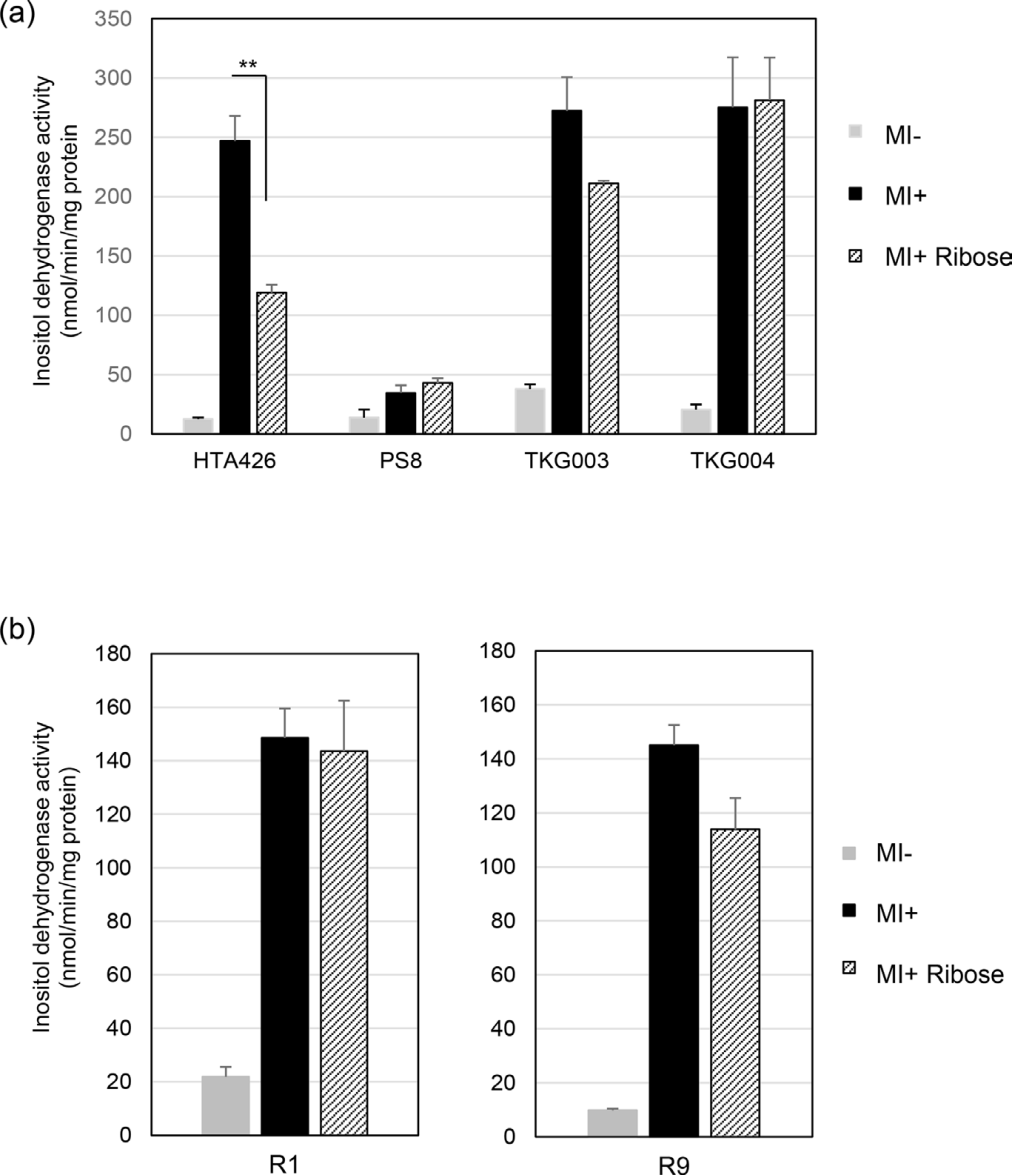
Inositol dehydrogenase activities in strains HTA426, PS8, TKG003 (HTA426 without *hprK*) and TKG004 [PS8 without *hprK*(G268R)] (a) and R1 and R9 (b) grown on various carbon sources. The mean±sd of three independent measurements is shown.

Next, to confirm whether HPrK is essential for ribose-induced catabolite repression of the *iol* operons, we measured inositol dehydrogenase activity in a variant (TKG003) of HTA426 with a deletion of *gk3082* (*hprK*) (Fig. 7a). The extent of ribose-induced repression in the presence of MI was reduced in TKG003 but not completely abolished. In contrast, a variant of PS8 (TKG004) with a deletion in *hprK*(G268R) exhibited no ribose repression, and inositol dehydrogenase activity was virtually identical in the presence of MI, irrespective of the presence of ribose.

To clarify the contribution of Crh to ribose-induced repression, we examined the extent of repression in two suppressor mutants predicted to produce little or no Crh (Fig. 7b). Suppressor mutant R1 carries a mutation in the start codon of *gk3063* (*crh*), and R9 carries a mutation in its putative ribosome-binding site, both of which are expected to impair *crh* translation. Consistent with these lesions, ribose-induced repression was abolished in R1 and markedly reduced in R9. These results indicate that Crh is required for ribose-induced CCR. Given that a slight suppression was observed in R9, it is speculated that some Crh might be produced despite the ribosome-binding site mutation.

**Fig. 8.**
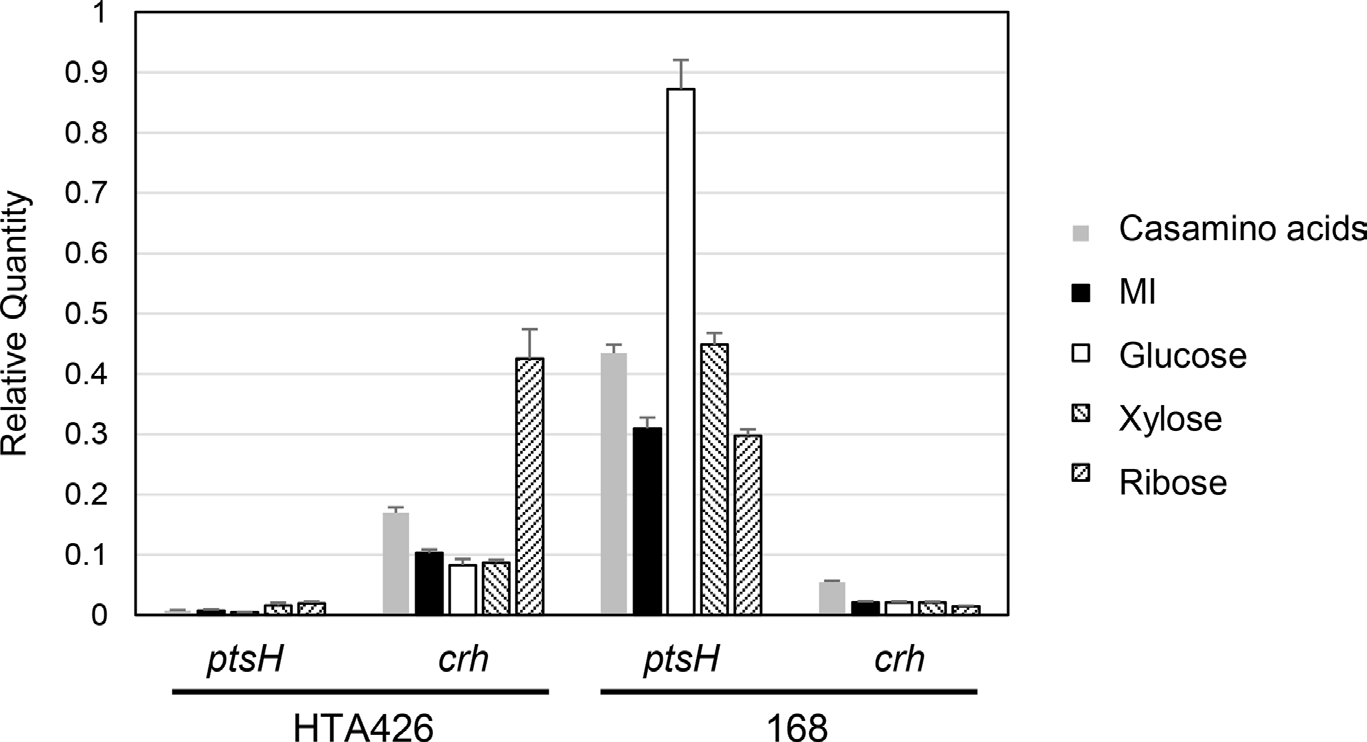
Transcription levels of *ptsH* and *crh* in strains HTA426 and 168 grown on various carbon sources. The mean±sd of three independent measurements is shown.

To investigate the differences between *G. kaustophilus* and *B. subtilis*, we measured the transcription levels of *gk3063* (*crh*) and *gk0995* (*ptsH*) in *G. kaustophilus* and *crh* and *ptsH* in *B. subtili*s grown in the presence of various carbon sources (Fig. 8). In *G. kaustophilus*, the transcription levels of *gk3063* (*crh*) were higher than those of *gk0995* (*ptsH*) regardless of carbon sources, with differences of ~5–20-fold. It should be noted that transcription of *crh* was particularly induced to a higher level by ribose. In *B. subtilis*, transcription levels of *ptsH* were consistently higher than those of *crh*. The transcription of *ptsH* in *B. subtilis* was found to be particularly induced in the presence of glucose. These results suggest that Crh might be the primary factor in the CCR of *G. kaustophilus*, while HPr serves this role in *B. subtilis*. These bacteria might have deployed the HprK phosphatase/kinase differentially in response to environmental carbon sources: Crh is phosphorylated in *G. kaustophilus* in response to ribose, whereas HPr is phosphorylated in *B. subtilis* in response to glucose.

### CcpA is required for the ribose-induced repression of *iol* operons in *G. kaustophilus*

As described above, the *iol* operons of *G. kaustophilus* undergo ribose-induced catabolite repression via Crh phosphorylation by HPrK. However, it remains to be confirmed whether phosphorylated Crh cooperates with CcpA, which functions in various Gram-positive bacteria as the component that binds to the chromosome DNA containing the nucleotide sequence of the *cre* element [28].

**Fig. 9.**
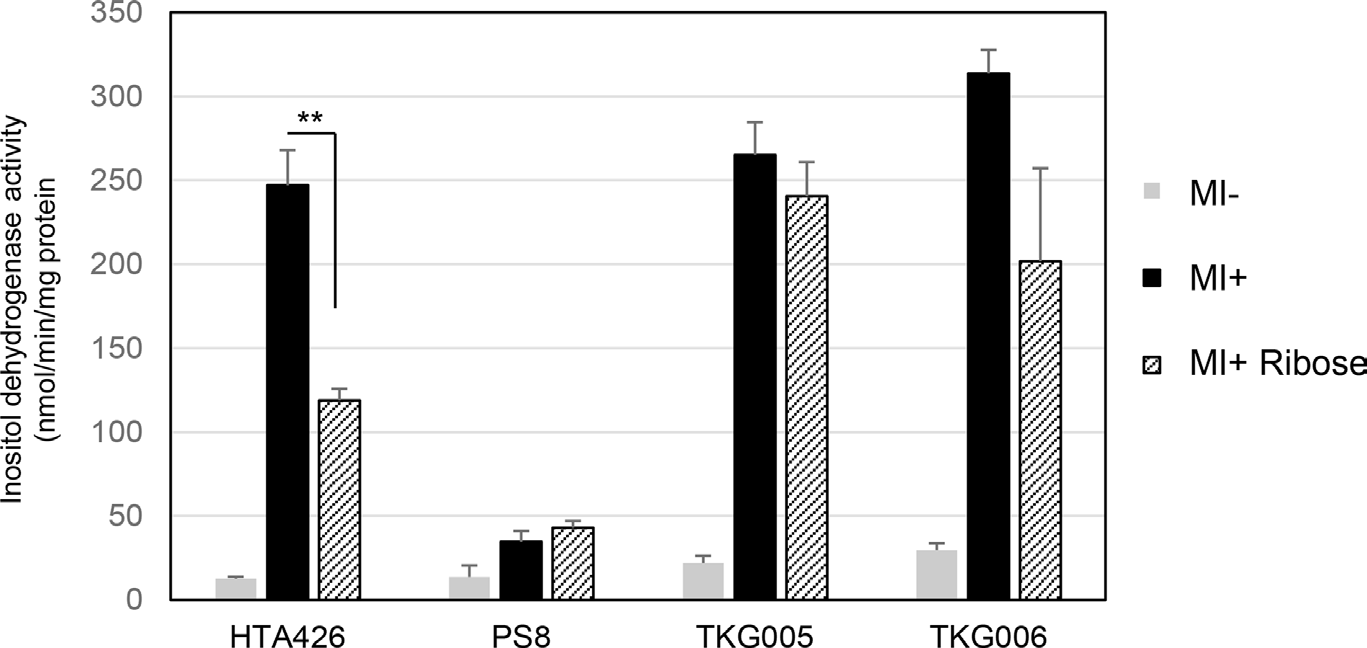
Inositol dehydrogenase activities in strains HTA426, PS8, TKG005 (HTA426 without *ccpA*), and TKG006 [PS8 without *gk2810* (*ccpA*)] grown on various carbon sources. The mean±sd of three independent measurements is shown.

Therefore, we examined inositol dehydrogenase activity in mutants with the deletion of *gk2810* (*ccpA*) (Fig. 9). In PS8, the *ccpA* deletion restored the induction of enzyme activity upon addition of MI. Furthermore, in both HTA426 and PS8, the *ccpA* deletion relieved repression of the enzyme activity in the presence of ribose. These results suggest that in *G. kaustophilus*, *ccpA* may be epistatic to *hprK* and *crh*, and CcpA is an essential factor that might cooperate with phosphorylated Crh to mediate the ribose-induced catabolite repression of the *iol* operons.

## Discussion

*G. kaustophilus* can utilize MI as a sole carbon source, and the genes involved in its metabolism are induced during growth on MI but repressed when ribose is co-present. Strain PS8, containing the *hprK*(G268R) allele, is unable to utilize MI as a sole carbon source, presumably because it accumulates phosphorylated Crh that leads to the constitutive repression of the *iol* genes. The inclusion of a plasmid-based copy of wild-type *hprK* into strain PS8 led to the release of constitutive repression of the *iol* operons, indicating that HprK was able to override the mutated function of the HPrK(G268R). Moreover, the inclusion of *hprK*(G268R) on another plasmid into the wild-type HTA426 strain led to the repression of the *iol* operons, like that of PS8, indicating that the additional copy of HprK(G268R) was able to override the function of the endogenous HPrK. We isolated seven suppressor mutants of PS8 that regained the ability to induce the *iol* operons and use MI as a sole carbon source. These suppressors had mutations in the ribosome-binding site or the start codon of *gk3063* (*crh*), either preventing or reducing its translation. These observations need to be confirmed biochemically by comparing the phosphorylation levels of Crh in the presence of HPrK and HPrK(G268R) *in vitro*. In addition, we need to clarify the differences in the amounts of Crh in PS8 and its suppressor mutants. It is also important to identify the chromosomal sequences at which the P-Ser-Crh and CcpA complex binds, as they may not be the same as the *cre* elements in *B. subtilis*.

In Gram-positive bacteria, HPr and Crh are factors with overlapping functions that are phosphorylated by HprK to form a complex with CcpA, exerting the transcriptional CCR. The reason for this overlap was unknown. In *G. kaustophilus*, regardless of the available carbon source, the expression of *gk3063* (*crh*) is higher than that of *gk0995* (*ptsH*) (Fig. 8). Notably, the addition of ribose increased the transcription of *gk3063* (*crh*) (Fig. 8), and the underlying mechanism for this induction remains to be determined. In contrast, we observed that in *B. subtilis*, *ptsH* expression exceeded that of *crh,* regardless of the carbon source. It was previously reported that in *B. subtilis*, glucose increases *ptsH* expression, resulting in strong CCR [26]. Carbon sources that induce strong CCR, such as glucose, are primarily taken up via PTS, involving phosphorylation of the His residue of HPr, but carbon sources that induce weak CCR, such as ribose, are not taken up by PTS and thus are independent of HPr [25]. The finding that ribose and arabinose induce partial CCR in *B. subtilis* represents a novel observation in this study (Fig. 5). In *B. subtilis*, metabolism of PTS carbon sources is normally prioritized, and HPr, which is central to PTS sugar uptake, also plays a major role in mediating CCR. In *G. kaustophilus*, however, Crh, which is not involved in PTS, may function in place of HPr to mediate CCR in response to ribose and xylose, whereas HPr appears to contribute little to this process. Although Crh is expressed at low levels in *B. subtilis*, it cannot be excluded that Crh may participate in partial CCR triggered by ribose or arabinose. An alternative possibility is that metabolism of ribose or arabinose does not generate sufficiently high levels of FBP in *B. subtilis*, resulting in inadequate phosphorylation of HPr. This inference might be related to the fact that repression by ribose and arabinose was partial in *B. subtilis*. These possibilities remain to be verified experimentally.

**Fig. 10.**
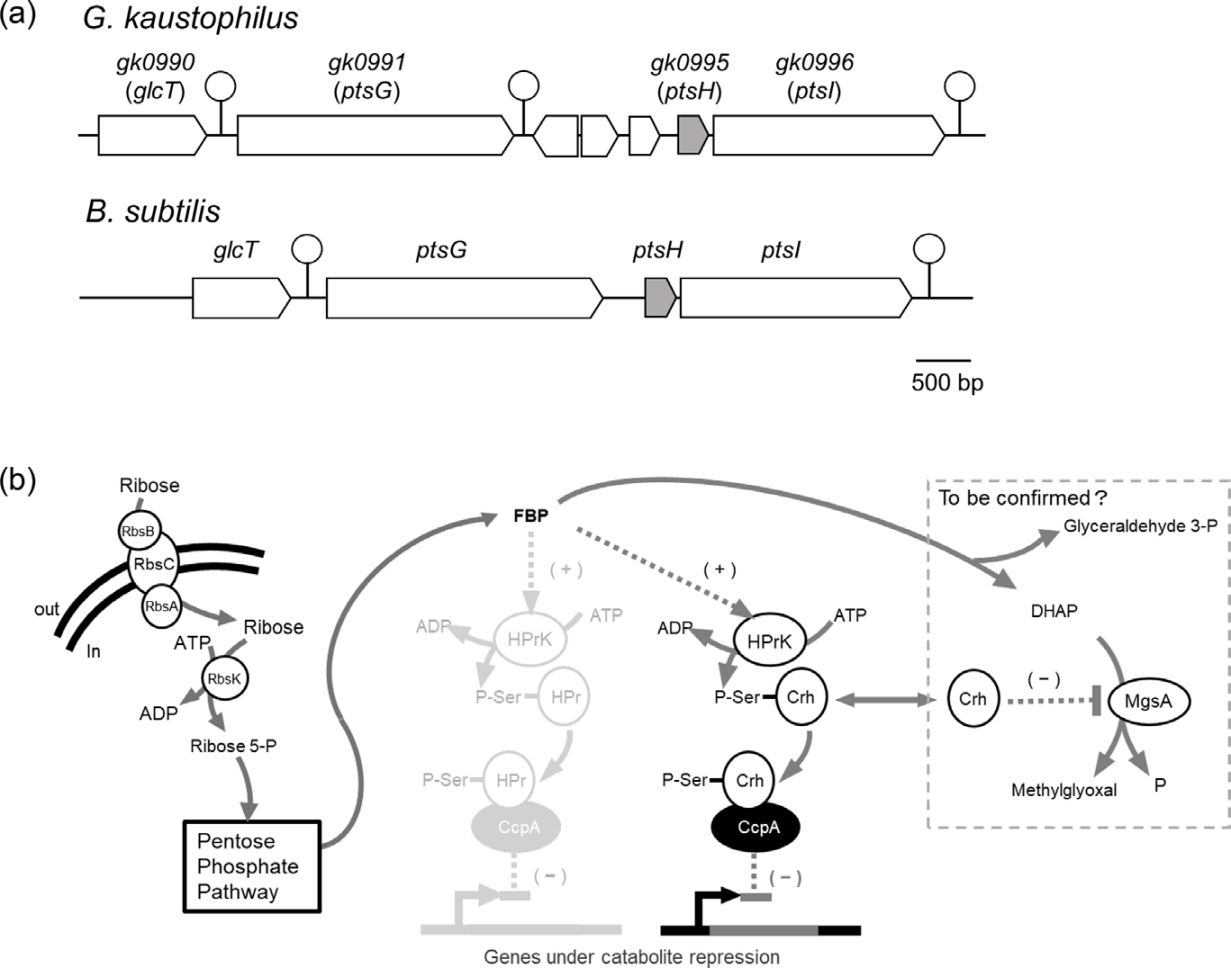
Comparison of gene organizations around *ptsH* in *B. subtilis* and *G. kaustophilus* (a) and the proposed mechanism for atypical catabolite repression in *G. kaustophilus* (b). The pathway involving Hpr, which is faded, is thought to be largely non-functional in *G. kaustophilus*.

In *G. kaustophilus*, which exhibits lower levels of *gk0995* (*ptsH*) expression, PTS carbon sources such as glucose are not taken up rapidly and do not induce CCR. In contrast, the higher levels of *gk3063* (*crh*) expression might facilitate CCR by non-PTS carbon sources, such as ribose. The differences in the *ptsH* expression levels between *G. kaustophilus* and *B. subtilis* may be attributed to differences in their transcriptional organization (Fig. 10a). In *B. subtilis*, *ptsG* and *ptsH*, which are involved in glucose PTS, are adjacent. When glucose is taken up, GlcT inhibits the formation of the terminator upstream of *ptsG*, thereby increasing transcription of the *ptsG* [29]. This, in turn, is thought to increase the transcription of *ptsH*. In *G. kaustophilus*, three genes and a transcription terminator are located between *gk0995* (*ptsH*) and *gk0991* (*ptsG*), meaning their transcription is not contiguous. These different gene organizations might be relevant to the disparity in expression levels of the *ptsH* between the two organisms.

When inositol dehydrogenase activity was measured using a strain deficient in both the ribose transporter and ribokinase, the ribose-mediated repression disappeared. After being taken up into the cell by the transporter, ribose is catabolized via the pentose phosphate pathway, yielding glyceraldehyde 3-phosphate (G3P) and fructose 6-phosphate. Fructose 6-phosphate is converted to FBP by phosphofructokinase. Furthermore, G3P accumulation also leads to increased FBP levels [30]. Therefore, it is likely that the increase in FBP levels from ribose metabolism could activate HPrK to enhance the phosphorylation of Crh. However, this possibility must wait for metabolome analyses to catalogue the relevant intracellular metabolites and their fluctuations in response to different carbon sources. This should also provide evidence to test our observation about whether a similar pattern of repression occurs for genes responsible for other carbon sources, in addition to the *iol* operons. Furthermore, it may offer insights into why ribose, rather than glucose, induces such extensive metabolic regulation.

Glucose, as the primary product of photosynthesis, constitutes the most abundant carbon source on Earth. It forms starch, cellulose and glycogen when polymerized. Consequently, glucose is the most readily available carbon source, and it occupies a central position in carbon metabolism not only for bacteria but for all other biological systems. The general cellular carbon metabolism network is based on glucose metabolism, and CCR has evolved to ensure that the most efficient carbon source is metabolized first. In this study, however, we observed that *G. kaustophilus* utilized ribose more efficiently than glucose, and the presence of ribose rather than glucose led to the repression of the *iol* operons. Thus, in this bacterium, activation of the CCR is atypical (Fig. 10b). We propose that in *G. kaustophilus*, an intermediate of ribose metabolism, presumably FBP, enhances the HprK-mediated phosphorylation of Crh, and the phosphorylated Crh forms a complex with CcpA that represses the transcription of *iol* operons. This is different from the situation in *B. subtilis*, where HPr performs that role. In the mutant strain Δrbs defective in ribose uptake, xylose-induced CCR still occurred (Fig. 5b). Therefore, it is unlikely that xylose and ribose uptake depend on the same system as reported for *Parageobacillus thermoglucosidasius* DSM 2542 [31]. Furthermore, the signal molecule promoting Crh phosphorylation could originate from a pathway common to both ribose and xylose metabolism. Nevertheless, whether the signal molecule is FBP remains to be clarified by future research. Alternatively, the signal might be a compound derived from the early stages of pentose metabolism.

There is a need to determine whether the atypical CCR observed for inositol metabolism applies to other carbon sources, and why *G. kaustophilus* prefers C5 carbon sources over C6 carbon sources: in particular, ribose is a carbon source for fundamental biological building blocks (e.g. nucleic acids, ATP and so on) rather than a primary energy source, such as glucose. On the other hand, the PTS appeared to be possibly less functional in glucose catabolism in *G. kaustophilus*, and in fact, it utilizes glucose more slowly. It is conceivable that glycolysis might be less active in this bacterium compared to others. Some bacteria prefer alternative pathways for glucose catabolism, such as the Entner–Doudoroff pathway, which involves fewer enzymatic reaction steps, rather than the common Embden–Meyerhof pathway. This suggests that these organisms may have evolved alternative strategies as a trade-off between energy yield and protein cost [32]. *G. kaustophilus* might have selected ribose as its preferred carbon source for some reason related to balancing costs and rewards. It is also interesting to consider whether this characteristic of carbon source metabolism is common in other bacterial species, particularly in relation to environmental preferences, including thermophilicity. Some enzymes or metabolic intermediates in glycolysis might be unstable in higher-temperature environments. In response to these questions, we plan to investigate the extent to which this phenomenon is found in other bacteria. The above observations not only challenge conventional wisdom regarding CCR in Gram-positive bacteria but also point towards potential applications, such as the proactive and proper use of thermophilic bacteria for their metabolic engineering.

## Abbreviations

CcpA: catabolite control protein A
CCR: carbon catabolite repression
Crh: carbon-flux-regulating HPr
5DG: 5-deoxy-d-glucuronic acid
DHAP: dihydroxyacetone phosphate
DKGP: 2-deoxy-5-keto-d-gluconic acid 6-phosphate
EI: enzyme I
EII: enzyme II
EMS: ethyl methanesulphonate
FBP: fructose-1,6-bisphosphate
5-FOA: 5-fluoroorotic acid
G3P: glyceraldehyde 3-phosphate
G6P: glucose 6-phosphate
2KMI: 2-keto-*myo*-inositol
MI: *myo*-inositol
MSA: malonic semialdehyde
PEP: phosphoenolpyruvate
PTS: phosphotransferase system
